# Genetic and Environmental Architecture of Ram Fertility Traits: A Review

**DOI:** 10.3390/genes17020210

**Published:** 2026-02-09

**Authors:** Kaiyue Zheng, Krishani Sinhalage, Guilherme Henrique Gebim Polizel, Ángela Cánovas

**Affiliations:** 1 Centre for Genetic Improvement of Livestock, Department of Animal Biosciences, University of Guelph, 50 Stone Road East, Guelph, ON N1G 2W1, Canada; kzheng04@uoguelph.ca (K.Z.); ksinhala@uoguelph.ca (K.S.); gpolizel@uoguelph.ca (G.H.G.P.); 2 Department of Animal Science, Faculty of Animal Science and Food Engineering, University of São Paulo, Av. Duque de Caxias Norte, 225, Pirassununga 13635-900, SP, Brazil

**Keywords:** breeding, candidate genes, management, scrotal circumference, ram fertility, sheep

## Abstract

Background/Objectives: Ram fertility is essential for sheep production, influenced by genetic, physiological, behavioral, and environmental factors. This narrative review synthesizes findings from over 190 peer-reviewed publications to evaluate the phenotypic indicators, genetic architecture, molecular candidates, and management conditions influencing testicular development, semen quality, and reproductive performance in rams. Methods: A narrative synthesis of peer-reviewed studies was conducted, integrating findings from quantitative genetics, genome-wide association studies, transcriptomics, and controlled environmental and management experiments. Emphasis was placed on studies evaluating fertility-related traits across breeds, ages, and production systems. Results: Recent genomic and transcriptomic studies have identified potential biomarkers (e.g., *IGF1*, *IGFALS*, *FOXO1*) and gene networks linked to ram fertility, including semen quality, scrotal circumference, and endocrine regulation. For instance, genome-wide association studies (GWASs) have identified candidate genes such as *SLC2A8* and *MAPK3*, which are associated with spermatogenesis and semen quality. Additionally, Y-linked SNPs such as *ZFY16*: g.146 C > T have been linked to testicular development. Genetic potential is heavily modulated by environmental constraints. Heat stress emerges as a disruptor of testicular thermoregulation, with recent evidence highlighting the vulnerability of spermatogenesis even in adapted breeds. Management interventions, specifically nutritional supplementation and hormonal modulation via melatonin, are discussed as effective strategies to mitigate environmental impacts. Conclusion: Improving ram fertility will require an approach that prioritizes phenotypic traits supported by candidate genes identified through transcriptomic analyses and GWASs. Integrating these genetic tools together with cost-effective nutritional and hormonal management strategies can further improve semen quality, libido, and testicular traits, thereby enhancing fertility gains while maintaining sheep breed adaptability across production systems.

## 1. Introduction

Ram fertility is a critical factor of reproductive efficiency and overall productivity in sheep production systems. Fertility refers to a ram’s ability to successfully impregnate ewes under defined mating conditions. Reduced fertility in rams can negatively affect flock productivity, lambing rate, and genetic progress. Several phenotypic traits are commonly used to evaluate ram reproductive potential, either individually or as part of breeding soundness examinations. These include external testicular measurements such as testicular length, width, and weight, scrotal circumference (SC), libido scores, and semen characteristics including volume, concentration, motility, and morphology. Studies have consistently linked testicular weight to reproductive performance, establishing a relationship with daily sperm production and an association with fertility [1,2,3,4,5,6,7,8,9].

Ram fertility could also be inferred from the reproductive outcomes of the ewes [10], which is also called fecundity, commonly expressed as conception rate (the proportion of mated ewes that become pregnant), pregnancy rate (the proportion of exposed ewes diagnosed as pregnant), lambing rate (the proportion of exposed or mated ewes that lamb), or litter size (the number of lambs born per lambing ewe) [11]. Selection for rams with high mating success could reduce the number of rams required for mating (lower ram-to-ewe ratios) and increase overall genetic gain, as ram mating success defined as the number of lambs sired by an individual is a heritable trait (h^2^ = 0.3) and is repeatable across mating seasons [12]. Sexual behavior involves precopulatory and copulatory activities directed toward estrous ewes, whereas libido reflects mating motivation, which is highly repeatable and exhibits moderate heritability (h^2^ = 0.22 ± 0.04); serving capacity represents the number of effective matings achieved under standardized conditions [13,14]. Although related, these measures capture distinct aspects of male reproductive performance. While libido correlates with the frequency of precopulatory behaviors, serving capacity more directly predicts flock fertility and is often used to identify high-performing sires in breeding programs [14].

Although farmers often prioritized appearance traits when selecting rams, many of the rams they prefer also exhibit high estimated breeding values (EBVs) for fertility traits, supporting a combined approach to selection [15]. To enhance the accuracy of selection for fertility-related traits, EBVs are widely applied in sheep genetic improvement programs [15]. EBVs provide a prediction of an individual’s genetic merit based on performance, pedigree, and statistical models such as multi-trait individual animal models [15,16]. Meanwhile, efforts have been made to identify candidate genes associated with testicular development, spermatogenesis, and semen quality. In Sanjabi rams, leptin gene polymorphisms were associated with testis weight and sperm morphology [3]. *IGF1* (insulin-like growth factor 1) polymorphisms influenced semen quality traits, such as motility and concentration [17]. Transcriptomic studies by Guan et al. [18,19] identified gene co-expression modules responsive to nutritional restriction in 24 Merino rams and found that key genes within these modules, including *RXFP1* (relaxin/insulin-like peptide receptor 1), *CFLAR* (*CASP8*- and *FADD*-like apoptosis regulator), and *NFKBIL1* (NF-κB inhibitor-like 1), were correlated with testis weight and spermatogenic activity in sheep. More recently, Pei et al. [20] identified Y-linked single nucleotide polymorphisms (SNPs) in *ZFY* and *EIF2S3Y2* that were significantly associated with SC and testicular weight in Suffolk and Hu rams, respectively, which provide potential as early molecular markers for ram fertility selection.

In addition to genetic control, ram fertility is influenced by breed, environmental conditions, and management practices [21,22,23,24,25,26,27,28]. Factors such as seasonality, nutritional status, stress, and social interactions can alter semen quality and reproductive behavior [4,21,29,30,31]. For instance, melatonin supplementation, which simulates short-day photoperiod conditions, has been shown to increase testicular volume in seasonally inactive rams or during non-breeding season [32,33]. Moreover, undernutrition can impair testicular development, partly through increased sperm DNA damage and apoptosis [19]. Therefore, hormonal and nutritional interventions, photoperiod manipulation, and body condition management are essential tools to enhance testicular development and semen quality, and, consequently, improve ram fertility.

In this literature review, we have examined phenotypic traits associated with ram fertility and the factors influencing reproductive performance by discussing common phenotypic traits including testicular traits, semen characteristics, and mating behavior. Moreover, candidate genes and key regulatory markers, followed by environment and management factors that affect fertility, such as photoperiod, thermal stress, nutritional inputs, common diseases, and social conditions were explored. These perspectives provide an integrated framework to understand and enhance reproductive efficiency in rams under diverse breeding programs.

### 1.1. Search Strategy and Information Sources

This narrative review used targeted searches of PubMed, Scopus, and Web of Science to identify literature on ram fertility. The searches, last conducted in December 2025, focused on phenotypic traits, quantitative genetics, molecular markers, and environmental or management factors, with emphasis on original English-language research published primarily from 2010 onward. Reference lists of key articles were manually screened, and non-ovine evidence was included only as supportive background. As a narrative review, study selection was based on relevance rather than a formal systematic protocol, and potential selection bias is acknowledged.

### 1.2. Keywords and Search Scope

To address the multidisciplinary scope of ram fertility, database searches combined the core terms “ram” AND “sheep” with topic-specific keywords corresponding to the review’s main themes. These included phenotypic traits, quantitative genetics, genomic and molecular markers, and environmental or management factors (e.g., testicular traits, EBVs, gene expression, GWASs, heat stress, photoperiod, nutrition, and disease).

### 1.3. Study Selection Rationale and Limitations

Studies were selected based on relevance to physiological, genetic, and environmental determinants of ram fertility, with research focused primarily on semen storage technologies excluded. The included studies provided empirical data on reproductive traits, reproductive physiology, or genomic associations, resulting in a final synthesis of more than 190 articles. As a narrative review, limitations include restriction to English-language publications and variability in terminology and trait definitions, which may affect literature retrieval. Differences in breed representation reflect the uneven distribution of available studies rather than deliberate prioritization.

## 2. Phenotypic Traits Related to Fertility

Evaluating phenotypic traits associated with fertility is crucial for identifying reproductively sound individuals in breeding programs. This section reviews commonly assessed phenotypic traits in ram fertility studies, including testicular characteristics, semen parameters, and the mating behavior of rams and the associated ewe fertility outcomes.

### 2.1. Testicular Traits

Testicular development is a key phenotypic trait in the evaluation of ram fertility [34]. Traits such as testicular weight, volume, length, width, and scrotum size are widely used to estimate the size and maturity of the testis. These measurements reflect the amount of seminiferous tissue present, which directly relates to the testis capacity for spermatogenesis occurring within the seminiferous tubules. Testicular size not only reflects structural maturity but also predicts sperm production efficiency. Positive associations between testicular length, width, and volume with semen volume, sperm mobility, and concentration were reported in Sanjabi rams [3].

**Figure 1:**
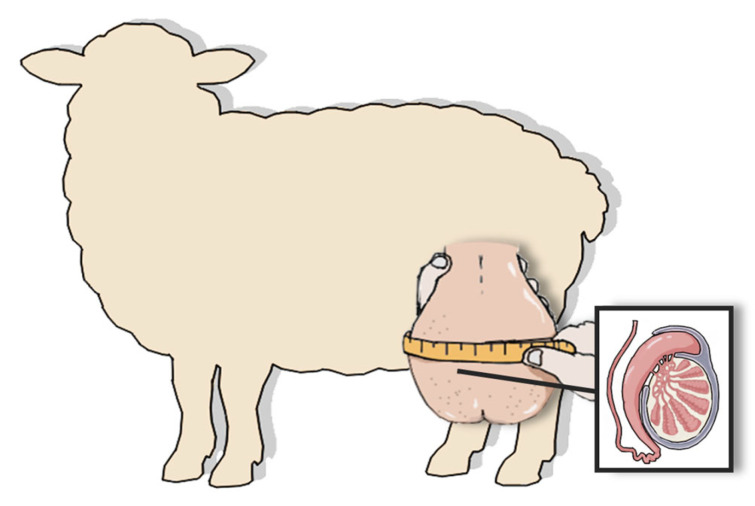
Illustration of scrotal circumference (SC) measurement in a live ram. The SC is recorded at the widest point of the scrotum using a flexible measuring tape with the testes gently held at the bottom. Created in BioRender, Kaiyue Zheng, (2025), https://www.biorender.com.

While testis weight is considered the most accurate predictor of daily sperm output and fertility [35], it cannot be measured in live animals [1,34]. As a result, indirect methods based on external dimensions such as SC and calculated testicular volume have been adopted as practical alternatives [3]. SC is a linear measurement of the scrotum and is used as a non-invasive estimate of testicular size. For instance, SC is measured in rams by firmly pushing both testis to the base of the scrotum and using a flexible tape to record the maximum circumference (Figure 1) [36]. Figure 1 demonstrates a non-invasive, quick, and reliable measurement of SC, which also serves as a proxy for assessing testicular size or weight [1]. In cases of dense scrotal wool or debris, the tape should be placed as close to the skin as possible to ensure accuracy [1]. The measurement of SC reflects the development of seminiferous tubules and the overall capacity for sperm production, which makes it a key phenotypic trait in ram fertility evaluations [37]. Testicular size in Turcana Alba rams increased chronologically, which is similar to SC increasing over time, likely because of sexual maturation [5,38]. Additionally, SC is a highly heritable trait and serves as a reliable indicator of sperm production in rams and for measuring breeding ability [25,39]. As Zamiri et al. [25] reported, SC was positively correlated with sperm concentration (r = 0.56) and negatively with sperm abnormalities (r = −0.70). However, Katahdin rams, in Mexico, with the largest SC among breeds studied, a showed lower sperm concentration [40], which reflects breed differences. Additionally, Zulu sheep, in South Africa, showed an insignificant positive correlation between semen volume and SC (r = 0.006) [38].

Standardizing procedures, such as the timing of measurement, whether rams are measured live or post-mortem, animal positioning, testis handling, and operator consistency, improves repeatability and minimizes measurement error. Normative values serve as important references in ram selection for SC. For the Gotlandic breed, a Swedish sheep population, normative SC values are 28.9 ± 1.9 cm in ram lambs (5–6 months old, 53.5 kg) and 32.4 ± 2.2 cm in mature rams (17–54-month-old) [36]. In Barbarine rams raised under Mediterranean conditions, the SC of untreated rams showed an increase from 26.7 ± 1.9 cm (at 16 months of age and approximately 55 kg body weight) to 29.5 ± 1.0 cm over 60 days [41]. In Hu-breed ram lambs, a Chinese meat breed known for early sexual maturity and high fecundity, Han et al. [42] reported that SC increased from approximately 11–12 cm at 56 days of age to 21–23 cm by 98 days in the control group. In terms of the relationship between SC and ewe fertility, the average ewe fertility increased as SC increased [43]. Aké-Villanueva et al. [4] reported that adult rams exhibited larger testicular measurements, improved semen quality, and enhanced mating performance compared to younger rams.

Although SC is moderately to highly heritable, several variations in SC should be considered for its adoption in industry, such as measurement variability [1], environmental (seasonal) fluctuations [25,26,44], and age and breed effects [4,40,45]. Even though many studies have tried to minimize the effect of measurement errors, which often requires the same technician across trials, SC can be influenced by external factors during measurement, such as SC covering with heavy wool and plant material [1]. SC also showed variances over time. The SC of Iranian fat-tailed rams reached peaks from October to March and were lowest during summer, which is likely associated with testosterone levels [25,26]. Similar findings were observed in Corriedale and Merino rams in Uruguay [46]. In terms of age, SC showed significant differences between ages 1–2 and ages 3–4 [38,45], reaching its peak at the age of 4 years and stabilizing thereafter [45]. Older Boujaâd rams (age: 5.5 to 6 years) showed larger SCs than the younger rams (age: 2.5 to 3 years) [44]. In the same study, semen quality was reported to be lower in older rams [44]. Furthermore, SC showed variations across breeds, which may be due to differences in weight and body size. There is also a highly positive correlation between body weight and SC (r = 0.7) [38,45]. The Dorper and Katahdin breeds have larger body sizes and were found to have higher SCs compared to Blackbelly and Pelibuey rams [40]. Ake-Villanueva et al. [4] hypothesized that testicular shape differences would result in SC differences across breeds because of elongated or round testicular shapes. SC tended to be larger in a bull with a round-shape testicle [47,48], which may explain why Dorper and Katahdin rams have higher SCs than Pelibuey and Blackbelly rams [4].

### 2.2. Semen Traits

In addition to testis, semen characteristics are commonly evaluated as phenotypic indicators of ram fertility. While semen evaluation does not always accurately predict actual fertility, it provides essential information for selecting males with high reproductive potential [1]. Semen is typically collected using an artificial vagina, a device that mimics the temperature and pressure of the female reproductive tract and is often used in fertility evaluations to assess ejaculate volume and quality [4,49,50,51]. Electroejaculation is another semen collection method employed widely in research [30,49]. Electroejaculation is faster and more convenient than using an artificial vagina, as it requires no habituation period, which allows for semen collection from many animals and yields a higher semen volume due to a greater proportion of seminal plasma [52,53].

The most-evaluated semen traits include semen volume, sperm concentration, motility (mass and progressive), viability, and morphology [51,54,55]. Semen volume represents the amount of ejaculate produced and is typically measured using a graduated tube [4] and is a standard semen trait measured routinely in artificial inseminations (AIs). Sperm concentration provides an estimate of the density of sperm cells within the ejaculate [30,50,56]. It is usually assessed using a hemocytometer [4] or photometric equipment [31,50]. Total sperm motility is another key trait, consisting of both mass motility, the collective movement of sperm in undiluted semen, and progressive motility, which refers to the proportion of sperm moving actively in a forward direction [4,50]. These traits are typically evaluated microscopically shortly after semen collection. In addition to motility, measuring sperm viability provides information on the proportion of live sperm within the ejaculate. Sperm viability is commonly assessed using vital stains such as eosin-nigrosin, which differentiate live from dead sperm cells [29,50,56]. Rams in the adequate-fertility group presented significantly higher sperm motility and viability compared to the low-fertility group (*p* < 0.05). Finally, sperm morphology, particularly sperm head, remains a standard component of semen analysis and continues to be widely used in breeding soundness evaluations and fertility screening [51,56,57]. Abnormal sperm head morphology in rams has been linked to reduced fertility, poor embryo quality, and early embryonic loss [57]. Almadaly et al. [58] also reported subfertile rams had significantly more abnormal spermatozoa. However, recent findings by Bodu et al. [55] found head and acrosome abnormalities were slightly higher in the adequate-fertility group (3.4%) compared to the low-fertility group (1.9%), while other defects (tail, midpiece) showed no difference. Sperm morphology in rams is often assessed via computer-assisted sperm head morphometry analysis (ASMA) to measure the cryopreserved sperm head traits including length (~8.08 μm), width (~4.80 μm), width-to-length ratio (~0.59), area (29.13 μm^2^), and perimeter (~23.93 μm), as reported in Gravance et al. [57]. Nuclear morphology has also been associated with fertility status, with elongated nuclear phenotypes linked to higher fertility (>82.4%), whereas deviations in roundness and circularity were associated with lower fertility [55]. In addition, seminal plasma profiles differ between fertility groups, with fertile rams showing distinct metabolic signatures, while subfertile rams exhibit elevated oxidative stress markers such as malondialdehyde [58].

Rams are classified as satisfactory if they produce at least 0.2 mL of semen with a sperm concentration greater than 1200 × 10^6^ sperm/mL, have a mass motility score larger than 2 (on a 0–5 scale, where 5 indicates the presence of dense and fast waves and 0 indicates no waves and no spermatozoa moving), and have a progressive motility greater than 60% [4,59,60].

Many studies have observed the effects of age and breed on the semen quality of rams. Abah et al. [61] summarizes that ram sperm quality peaks approximately at the age of three, then declines. Age group significantly affected sperm concentration, with young rams showing the lowest concentration (3133.8 ± 151.5 × 10^6^ sperm/mL) [4]. Similar findings were also reported in many studies [38,56,61,62]. Semen volume and total sperm peaked at age three and declined thereafter [38,62], with sperm abnormalities lowest at that age [38]. Age effects on motility are not consistent, with some reports showing minimal or non-significant changes during early sexual development. Ntemka et al. [54] indicated that semen motility and other functional traits (e.g., concentration, volume, morphology) were not limited by age in Chios rams, which could maintain high semen quality up to 13 years of age. No age influence was observed in Awassi rams [63]. Additionally, while semen volume, concentration and motility were not affected by season, some seasonal changes for breeding and non-breeding periods in sperm morphology may impact fertilization capacity [38,54]. However, findings on the association between age and sperm morphology have been inconsistent. [61].

Breed effects were found in semen traits [40,62]. For instance, Lacaune rams had better semen parameters overall than Manech Tête Rousse rams [62]. Dorper and Katahdin rams had lower semen quality (lower motility and higher abnormalities), while Pelibuey and Blackbelly rams showed better semen quality, including higher motility and lower sperm abnormalities, likely due to better environmental adaptation in tropical areas [40]. Aké-Villanueva et al. [4] reported that breed had no effect on any of the evaluated seminal traits, including semen volume, sperm concentration, mass motility, individual motility, and abnormal morphology in air sheep rams in Mexico.

### 2.3. Libido, Mating Behavior, and Ewe-Based Fertility Outcomes

Although libido and mating behavior show breed-specific variations due to factors such as season, photoperiod, and nutrition, libido may serve as an indicator of ram mating success [64]. Libido and mating behaviors reflect a ram’s sexual motivation, capacity to seek out and mount receptive ewes, and ability to complete successful copulations. Therefore, they are critical for ensuring fertilization, especially in extensive and semi-intensive sheep production systems, where rams need to cover multiple ewes. For example, Lecic et al. [10] reported mating results under a study design in which 8 rams were used to service 474 ewes. Many studies included behavioral tests as part of responses. Common behavioral indicators used to evaluate libido are the number of mounts, successful ejaculations, vulva sniffing, lateral approaches, and total activity duration. These traits are often scored during structured observations [4,41,50].

In addition to male performance, ewe-based fertility outcomes, such as pregnancy rate, lambing rate, and litter size, are often used as indirect measures of ram fertility. Cevik et al. [32] used pregnancy rate, twinning rate, and number of live offspring per ewe as metrics to compare the fertility potential of rams, which demonstrates ewe-based results for evaluating ram performance in natural service systems and provide insights on studies where direct semen evaluation may not be feasible. In the other research by Shorepy and Notter [2,65], a significant association was observed between male reproductive traits and the reproductive performance of their ewe mates. Specifically, rams with higher reproductive scores early in life sired offspring that demonstrated increased lambing rates and enhanced reproductive outcomes, suggesting a lasting impact of ram fertility on overall flock productivity. However, similar to ram behavioral responses, ewe performance also depends on breed and environment. Reproductive outcomes such as conception and lambing rates are influenced by the physiological and behavioral readiness of animals, which is shaped by both intrinsic traits and the production environment [66].

## 3. Phenotypic Correlations Among Ram Fertility Traits

Phenotypic correlations among observable fertility traits such as semen characteristics, testicular morphology, and sexual behavior provide information on the selection of rams for reproductive performance. Studies evaluating these relationships across breeds and age groups have revealed both positive and negative associations.

### 3.1. Semen Quality and Fertility Outcomes

Several studies have reported positive phenotypic correlations between semen traits and indicators of fertility in rams. David et al. [62], analyzing 62,946 ejaculates from Lacaune and Manech Tête Rousse rams, found strong positive correlations between ejaculate volume and the total number of spermatozoa (r = 0.85 to 0.89). A positive weak correlation was also observed between sperm concentration and motility (r = 0.18 to 0.23) and volume and concentration were weakly correlated (r = 0.01 to 0.13) [62]. Additionally, phenotypic correlations between motility and either ejaculate volume or total sperm count were low (r = –0.04 to 0.07), suggesting limited association between these traits [62]. Areb et al. [67] reported that six-month body weight in Bonga rams was positively correlated with sperm motility (r = 0.58) and concentration (r = 0.45) but negatively correlated with semen volume (r = −0.87) and total sperm count (r = −0.83).

### 3.2. Testicular Morphology and Semen Traits

Testicular morphology traits, SC, testis weight, and testicular dimensions, have demonstrated significant phenotypic associations with semen characteristics. In Merino rams, increases in SC were positively correlated with semen volume (r = 0.7), sperm concentration (r = 0.5), and total sperm number per ejaculate (r = 0.7) [19]. These findings are consistent with Areb et al. [67], who investigated 101 Bonga rams under a Community-Based Breeding Program (CBBP) in Ethiopia and reported in that SC was positively correlated with sperm motility (r = 0.54) and sperm concentration (r = 0.55). Similarly, six-month body weight was positively associated with SC (r = 0.83 ± 0.598) [67], suggesting that the finding of the strong positive correlation between SC and six-month body weight is consistent with other studies [68], indicating that this may be due to shared physiology development. Chella et al. [38] reported a positive correlation between SC and semen volume, even though it was not significant (r = 0.006, *p* >0.05).

### 3.3. Sexual Behavior and Semen and Fertility Traits

Although less frequently quantified, sexual behavior traits have also shown phenotypic association with semen quality and fertility outcomes. Pascal et al. [22] reported that rams with higher sexual behavior scores, based on mounting attempts and libido reflexes, had higher sperm motility, a greater proportion of live spermatozoa, and achieved higher conception rates and lambing success. While specific correlation coefficients were not provided, group-level comparisons demonstrated statistically significant improvements across both behavioral and seminal traits. These results suggest that behavioral evaluations may serve as useful phenotypic indicators of reproductive potential, especially when semen analysis is limited or not feasible in field conditions.

In another study, Moghaddam et al. [69] investigated the relationships among peripheral blood testosterone concentrations, SC, sexual behavior, and semen characteristics in 12 crossbred rams representing combinations of four breeds, Arkharmerino × Moghani, Baluchi × Moghani, Ghezel × Baluchi, and Ghezel × Arkharmerino. Moghaddam et al. [69] measured sexual behavior (libido) using six parameters, which were reaction time (RT) and time taken for both the first and second ejaculations, and the number of mounts (NOM) for each. The results showed that only the number of mounts for the first ejaculation (NOM1) showed a statistically significant difference among the four genetic groups (*p* < 0.05). Specifically, Baluchi × Moghani rams had a significantly higher NOM1 (6.0 ± 1.01) compared to the other groups. Although Ghezel × Baluchi rams had the highest mean testosterone (7.12 ng/mL), no significant difference in testosterone levels was detected across breeds. Additionally, testosterone was not significantly correlated with any libido or semen trait, suggesting that circulating testosterone is not a reliable indicator of reproductive performance. In contrast, SC was positively associated with sperm concentration (r = 0.739) and negatively correlated with reaction time for second ejaculation (r = –0.622), indicating SC as a proxy for spermatogenic and sexual function and that testosterone levels alone may have limited predictive power in crossbred rams [69].

## 4. Genetic Correlation of Ram Fertility Traits

There is limited research available on EBVs and genetic correlations specific to ram fertility traits, making this an underexplored but important area. Heritability and genetic correlations are essential for understanding the genetic control of ram fertility and guiding the use of EBVs in selection programs. Heritability estimates confirm that additive genetic variation exists for each ram fertility trait, while the strength is trait- and age-dependent. Current evidence suggests that semen traits generally exhibit low-to-moderate heritability, including semen volume, gross motility, concentration, and total sperm, which may be due to environmental variability such as complex traits related to fertility, nutrition, or management [67,70]. Ejaculate volume ranges from 0.07 at the age of 9 months to 0.11 at the age of 12 months in Horro and Menz ram lambs [71] and reaches 0.23 ± 0.12 in adult Bonga sires [67]. Gross motility is around 0.03 in Australian Merino AI rams [70]. An initial mass motility of 0.1± 0.012 reaches 0.31 ± 0.10 at the age of 6 months in Bonga sheep [67] and 0.32 ± 0.11 at the age of 9 months in Horro/Menz breeds, while sperm concentration is low-to-moderate at the age of 12 months (0.17 ± 0.15) in the same population of Horro and Menz rams [71]. Low-to-moderate heritability estimates were reported for ejaculate volume (0.08–0.20) and sperm concentration (0.10–0.19) across five Spanish dairy sheep breeds [72].

SC is consistently reported to be more heritable than semen quality traits, making it a reliable indicator for genetic selection to improve ram fertility. A synthesis of 165 studies from 1992 to 2003 reported a mean heritability of 0.21 ± 0.06 [73], which was higher than the heritability of any semen trait included in the same review article. In comparison, a previous review reported SC heritability estimates ranging from 0.08 to 0.50 with a mean of 0.24 ± 0.04 [74]. However, breed- and age-specific values should be considered. For instance, Bonga rams showed a lower SC value of 0.11 ± 0.16 (ages not specified) [67] compared to 0.25 ± 0.40 in the Merino breed [43]. Additionally, Iranian composite lambs recorded SC values of 0.31 ± 0.08 at 60 days and 0.41 ± 0.09 at 90 days of age [16]. In Hu lambs, SC heritability peaked at 0.72 ± 0.12 at 100 days and declined to 0.32 ± 0.12 by 140 days [75], which is consistent with the SC heritability estimates reported for Rambouillet rams, which ranged from 0.22 to 0.6 between 90 and 180 days of age [34]. Ma et al. [75] suggested that Hu sheep experience slower growth between 160 and 180 days of age and since SC is influenced by body weight, SC remains unchanged during this period. Structural sperm defects also carry moderate heritability. For instance, a heritability estimate for proximal droplets was 0.42 ± 0.19 at nine months of age, although the defect would decline as rams mature (0.30 ± 0.11 at 12 months) [71]. Overall, the SC heritability results from different studies highlight early-life SC (≤120 days) as a proxy that can be used in the breeding program.

In terms of genetic correlations, SC values in Bonga sires show strong positive genetic relationships with six-month body weight (0.83 ± 0.60), mass motility (0.54 ± 0.30) and sperm concentration (0.55 ± 0.14), whereas semen volume shows negative genetic correlations with most traits including motility, concentration, six-month body weight, and SC, except total spermatozoa, with which it has a positive correlation (0.66 ± 0.213) [67]. The estimates were derived from 101 rams from 30 sires and 39 dams, and the large standard errors may indicate uncertainty. In Horro and Menz rams, twelve-month SC is positively correlated with semen volume (0.55 ± 0.11), mass motility (0.62 ± 0.20), and individual motility (0.54 ± 0.12), but negatively correlated with abnormal sperm morphology (–0.75 ± 0.24). Mass motility and concentration are almost perfectly genetically correlated at 0.98 ± 0.22 [71]. Therefore, selecting on SC is expected to increase sperm concentration, motility, and normal morphology. However, the genetic correlations were estimated from a dataset of 278 Horro and Menz ram lambs based on 10 sires mated to 25 ewes [71], which limits the number of sire families contributing to the analysis. Together with the large standard errors reported, the breeding structure suggests that the correlation estimates should be interpreted with caution.

There are few available studies on cross-sex correlations. In a composite sheep population with Dorset × Finn × Rambouillet, ewe spring fertility shows r = 0.56 with fall litter size and 0.29 with SC measured at 90 days across 48 to 54 sire families [65]. Standard errors were not provided for SC values. A meta-analysis based on six studies across breeds reported a genetic correlation of 0.20 between ram SC and ewe fertility [73]. These values emphasize that estimates linking male and female reproduction traits are scarce and are needed to evaluate further when the breeding objective targets flock-level reproduction. Meanwhile, relationships between fertility and growth are context-dependent. In the Dorset composite flock, spring fertility is negatively genetically correlated with 90-day weight (–0.31), even though SC at 90 days shows a strong positive correlation (0.60) with the same weight [65]. In the selection line of a Dorset × Rambouillet × Finnsheep composite, fertility is negatively linked to birth weight (–0.37 ± 0.30) but has a weakly positive (0.09–0.15) correlation with weight recorded from 60 to 120 days [16]. Breeding indices must therefore weight growth traits carefully to avoid reducing realized mating success. Using DNA data from over 150,000 progenies in New Zealand flocks, Juengel et al. [12] reported low heritability of ram mating success (h^2^ = 0.04–0.06) but moderate repeatability (r = 0.23–0.32), indicating consistent performance across seasons. Genetic correlations with lamb growth and fecundity were not significant, suggesting that selection for mating success can reduce ram-to-ewe ratios without compromising production traits.

## 5. Estimated Breeding Values (EBVs)

According to Asadi-Fozi et al. [16], a composite sheep line derived from Dorset, Rambouillet, and Finnsheep breeds showed significant genetic improvement in fertility and SC over time. Spring fertility EBV rose from –0.022 to 0.15 between 1989 and 2005, with an average gain of 0.01 units per year, while SC EBV increased by 0.04 cm/year at 60 days, 0.09 cm/year at 90 days, and 0.07 cm/year at 120 days. Similarly, a fall-lambing composite flock achieved 0.014 units/year in fertility EBV over 6 years, with correlated gains of 0.032 cm per year in SC at 90 days and 0.0087 lambs per year in litter-size EBV [2].

Social factors also play a significant role in the sheep industry. In Ethiopia, farmers ranked rams primarily based on appearance traits (e.g., coat color, horns, tail type). However, these rankings showed only a weak correlation (ρ ≈ 0.20–0.30, where ρ is Spearman’s rank correlation coefficient) with scientific rankings based on growth EBVs. A two-stage system, selection on EBVs in the nucleus flock followed by farmer choice among the top sires, was proposed to integrate genetic gain with breeder preference [15]. Similar application will be critical for fertility-specific EBVs, whose benefits are not immediately visible.

## 6. Candidate Genes Associated with Ram Fertility

### 6.1. Transcriptomic Evidence Across Developmental and Mature Stages

Transcriptomic studies have helped identify the genes and key regulatory elements involved in testis development, spermatogenesis, and post-testicular sperm function in rams [76,77]. These studies generate large-scale gene expression data that allow researchers to identify differentially expressed genes (DEGs) and signaling pathways linked to fertility traits, including in non-ovine species [78,79,80,81,82,83]. Testis transcriptomes, in particular, offer a developmental map of how gene networks change as the reproductive system matures, which provides a foundation for selecting functionally relevant candidate genes [76,77].

Recent transcriptomic studies have provided valuable insights into the molecular mechanisms underlying ram fertility across both the developmental and mature reproductive stages (Table 1). Because several findings are breed- or study-specific, the associations reviewed here should be interpreted as context-dependent. Xi et al. [77] used strand-specific Illumina RNA-Sequencing (RNA-Seq, ≈60 M paired reads per library) to profile testicular gene expression of Southdown × Hu F1 rams at 0, 3, 6, and 12 months. They detected 2987 up- and 3964 down-regulated mRNAs (in the 6-month age group compared to the 1-year age group) and 2961 skipped-exon (SE) events. Gene Ontology (GO) and pathway enrichment analyses using the Kyoto Encyclopedia of Genes and Genomes (KEGG) database (http://www.genome.jp/kegg/, accessed on 21 July 2022) revealed that DEGs were involved in the cAMP, mitogen-activated protein kinase pathway (MAPK), Phosphoinositide 3-kinase–Akt pathway (PI3K-Akt), and Forkhead box O (FoxO) signaling pathways, key regulators of testis cord formation, germ cell development, and hormonal signaling during puberty. cAMP enhances sperm motility and testosterone production through Sertoli and Leydig cell activation, while MAPK regulates germ cell proliferation and differentiation. PI3K-Akt supports cell survival and metabolism in testicular cells, and FoxO ensures cellular homeostasis and germ cell maintenance. Upstream regulator analysis highlighted *GATA4*, essential for Sertoli cell function and testis formation, *SOX9*, an important factor in Sertoli cell differentiation and male sex determination, *SMAD4*, a mediator of Transforming Growth Factor (TGF)-β signaling and germ cell development, *YAP1*, which promotes proliferation of support cells via the Hippo pathway, *FOXO1*, which regulates cell cycle progression and survival of testicular cells, and *ITGB1*, an integrin involved in extracellular matrix–cell interactions during testis development. [77]. Additional genes with functions are provided in the Supplementary Table S1.

Furthermore, Xi et al. [77] demonstrated that alternative splicing (AS) has emerged as a key post-transcriptional mechanism regulating testicular development and function in rams. Using replicate Multivariate Analysis of Transcript Splicing (rMATS), five canonical AS types were identified across the following developmental stages: SE, intron retention (RI), mutually exclusive exons (MXE), and alternative 3′ and 5′ splice sites (A3SS, A5SS), with SE being the most common event. The frequency of AS events increased with developmental stage, especially during the transition from puberty (at about 6 months of age) to young adulthood (at 12 months of age), where 2961 SE events were detected compared to 1936 during the earlier prepubertal phase (birth to 3 months). This trend suggests that transcriptome complexity intensifies during late testicular maturation, likely to support the demands of full spermatogenic function. The gene *DDB1*, involved in Sertoli cell proliferation and testicular cord formation, exhibited AS at all stages (from birth to year 1), suggesting its central regulatory role during testis maturation.

In parallel, Xi et al. [84] performed small RNA-Sequencing of the same Southdown × Hu F1 rams to profile microRNA expression across developmental stages. They identified 787 known and 415 novel miRNAs, with differentially expressed miRNAs predicted to target genes such as *YAP1*, *SMAD4*, *SOX9*, and *DOT1L*. Functional enrichment showed that these miRNA–mRNA networks converge on the same pathways highlighted by the mRNA study, including MAPK, FOXO, Hippo, Wnt, and cAMP signaling. The authors noted that miR-34c, miR-21, and miR-499b were strongly expressed in year-1 adult testes, which are associated with spermatogenesis, Sertoli cell regulation, and testicular cell apoptosis (S1). The two studies by Xi et al. [77,84] showed that coding genes, alternative splicing, and miRNA regulation form complementary layers of control over fertility-related pathways in the developing ram testis.

In addition to the developmental transcriptomic analysis, Hitit et al. [76] applied ribosomal-RNA-depleted NovaSeq-6000 sequencing to ejaculated sperm from rams whose conception rates were 99% (i.e., high-fertility) or 74% (i.e., low-fertility), identifying 93 long non-coding RNAs (lncRNAs) and 275 mRNAs that differed between the two groups, which are associated with sperm motility, flagellum structure, and mitochondrial function. Hitit et al. [76] identified 141 up-regulated genes in high-fertility rams, many of which are associated with sperm structure and motility, particularly in KEGG and GO pathway enrichment analyses, including *ABHD2*, a lipase involved in sperm activation via progesterone signaling, *AK1*, an adenylate kinase important for ATP homeostasis, *CABS1*, a calcium-binding protein involved in calcium signaling, *ROPN1*, a structural component of the fibrous sheath, *TEKT3*, a tektin that stabilizes axonemal microtubules, *SEPTIN2*, a cytoskeletal organizer enriched in motile cilium and sperm flagellum pathways, as well as *CCDC39* and *TTC12*, which both contribute to axonemal assembly and flagellar function [76]. While Xi et al. [77] focused on gene expression dynamics in the developing testis, identifying pathways and transcription factors essential for spermatogenesis, and Hitit et al. [76] profiled ejaculated sperm, highlighting mRNAs and lncRNAs associated with sperm motility and fertility status, these studies provide complementary views of ram fertility from developmental regulation in the testis to functional gene expression in mature sperm.

Hormonal and nutritional regulation in the ram testis is influenced by gene expression networks, especially under dietary modulation [85]. High-grain-fed (HG) rams showed significantly higher body weight, testosterone, IGF-1, and growth hormone levels compared to hay-fed controls. HG diet increased the number of Sertoli cells, spermatogonia, and spermatozoa in seminiferous tubules, suggesting enhanced spermatogenesis, while testis weight and diameter were not significantly affected. Zhang et al. [85] studied Illumina HiSeq 3000 RNA-Seq and identified 59 differentially expressed lncRNAs and 229 mRNAs between HG rams and hay-fed groups during sexual maturation. GO and KEGG analysis revealed that enriched pathways and gene functions were related to spermatogenesis, sperm motility, steroid hormone synthesis, apoptosis, cAMP, MAPK and Erythroblastic leukemia viral oncogene B (ErbB), TGF-*β* and Hedgehog, and PI3K-Akt signaling, including that of *BMP2*, *RASGRF2*, *TSHZ2*, and *ESRRG*. Co-expression network analysis showed that multiple lncRNAs were closely correlated with reproduction-related genes [85]. For example, *PAPPA2*, which regulates the IGF system, was co-expressed with LOC105605113 (down-regulated) and LOC105609364 (up-regulated). *GABRP*, involved in paracrine signaling and testicular neuroendocrine regulation, was co-expressed with LOC106991431 down-regulatedd). Additionally, *KRT18*, which may help maintain the structural integrity of Sertoli cells or developing germ cells in the testis, was co-expressed with LOC105605281 up-regulatedd) and LOC106991234 (up-regulated). Another diet study emphasizes the importance of metabolic state. Guan et al. [18] performed poly-A-selected Illumina RNA-Seq on testis from feed-restricted Merino rams, revealing 2243 DEGs, 206 nutrition-sensitive alternative splicing events, and WGCNA modules 7, 9, and 10 that were linked to reduced SC to apoptosis regulators such as *NFKBIL1*, *CFLAR*, *MAP3K1*, and *MAP3K7*. Zhao et al. [86] combined Illumina RNA-Seq with untargeted LC–MS metabolomics after 72 days of L-citrulline supplementation. They recorded 961 DEGs in the hypothalamus (524 up, 437 down) and 715 testicular DEGs (260 up, 455 down). Network analysis highlighted hub genes in the hypothalamus (*GNRH1*, *PTPN11*, *KDR*) and in the testis (*JAK2*, *PRIM1*, *MCM6*). GO and metabolic pathway enrichment using the KEGG database revealed glycolysis/gluconeogenesis as a significantly enriched pathway in hypothalamic DEGs, indicating enhanced sperm energy metabolism through combined transcriptomic and metabolomic analyses [86].

Sexual behavior in rams appears to be regulated by multiple neuroendocrine networks, especially in the hypothalamus [87]. They investigated the molecular basis of sexual behavior variation in rams by comparing gene expression profiles in the hypothalamus, pars tuberalis, and pineal gland between sexually active and nonactive Rasa Aragonesa rams using RNA-Seq. A total of 103 DEGs were identified in the hypothalamus and 12 in the pars tuberalis, while no DEGs were found in the pineal gland. In active rams, up-regulated genes included *MTNR1A*, *AVP*, *PDYN*, *GNRHR*, *FSHB* and *LHB*, *TSHB*, *CGA*, and *GABRD*. These genes were enriched in GnRH, cAMP, and neuroactive ligand–receptor signaling pathways. Inactive rams showed downregulation of *NPY*, *MCHR1*, and *CRABP1*, which are linked to stress and behavioral inhibition. RT-qPCR confirmed the expression patterns, suggesting these genes play a key role in regulating ram sexual behavior and may serve as markers for reproductive selection [87].

Spermatozoa transcriptomes differ between sheep breeds, and sperm RNA profiles could be potential biomarkers for AI success. Hodge et al. [28] expanded the transcriptomic dataset across three breeds (Merino, Dohne, Poll Dorset) in Australia. There are some common genes across contrasts, with 41 DEGs shared in Merino vs. others, 31 in Poll Dorset vs. others, and 4 in Dohne vs. others. Some were linked to reproduction (e.g., *MAPK3*, *MED6*, *GTL2*), growth (e.g., *MKLN1*, *CHST4*, *LMBR1*), wool traits (e.g., *TGFB2*, *KRT4*, *IVL*), and testis-specific genes (e.g., *RNF151*). Thirty-nine DEGs differentiated high- from low-quality semen, such as *OXCT2*, involved in ketone body metabolism, which was down-regulated in low-quality semen samples [28].

### 6.2. Single Nucleotide Polymorphisms (SNPs) and Functional Assays

Targeted association studies reinforce the involvement of growth-regulating and structural sperm genes. The candidate genes identified in Section 6.2 and Section 6.3 are summarized in Table 2, with detailed functional descriptions with associated proteins and phenotypes presented in Supplementary Table S2.

Recent studies have demonstrated the relationship between specific gene polymorphisms and ram fertility traits in semen quality and fertility outcomes. Bakhtiar et al. [17] genotyped two exon-3 SNPs in the LEP gene (leptin, a metabolic hormone modulating energy balance and reproduction) using Polymerase chain reaction—restriction fragment length polymorphism (PCR-RFLP) in 96 Sanjabi rams. The AA genotype at g.170 G > A showed greater sperm motility, while the GG genotype corresponded to greater SC. At g.332 G > A, similar relationships were observed for sperm viability and testis size [3]. In the same breeds, Bakhtiar et al. [17] focused on the *IGF1* gene, a key regulator of cell proliferation and steroidogenesis. They identified SNPs in the 5′ flanking region and exon 3 that were significantly associated with sperm concentration, motility, and membrane integrity, but not testicular dimensions. The CT genotype in exon 3, for example, showed enhanced sperm output and a hypo-osmotic swelling response [17]. Similarly, Kianpoor et al. [88] examined polymorphisms in *CYP19* (P2 A113G) and *MTNR1A* (C893A) in 96 Sanjabi rams and reported targeted but limited effects on male reproductive traits. The *CYP19* promoter variant was associated with slightly larger SC measurements, while the *MTNR1A* SNP influenced sperm morphology. However, neither locus showed significant associations with sperm motility, concentration, viability, or other testicular measurements.

Another study reported *IGFALS* polymorphisms were associated with testicular development and increased SC by up to 1.1 cm in Hu lambs, supporting its role in endocrine growth modulation [75]. Similarly, the *FecXR* allele of *BMP15*, a TGF-*β* family gene essential for folliculogenesis, improved mass motility and the proportion of rapid spermatozoa in Rasa Aragonesa rams, although it did not significantly alter testicular size [6], particularly during winter. In contrast, Chen et al. [89] reported that *BMP15* expression in the ram is epididymis-specific and significantly higher in low-fecundity breeds (*p* < 0.01), suggesting a potential negative association with male reproductive efficiency. The same study showed that *GDF9* expression was detected across tissues but was higher in the vas deferens of high-fecundity Small-tail Han rams than in low-fecundity Sunite rams (*p* < 0.05), whereas the downstream Smad signaling components (*Smad1*, *Smad5*, *Smad9*) exhibited expression patterns more similar to *BMP15*, with elevated Smad1 in the vas deferens and Smad9 in the epididymis of low-fecundity rams, indicating tissue-specific regulation of the TGF-β/Smad pathway. *SPATA6*, a sperm-associated antigen involved in sperm head–tail linkage, harbored two SNPs (c.3631C > G in the 5′-UTR and c.937A > G in the 3′-UTR) that were significantly associated with increased epididymal weight in Hu rams [90]. The qPCR and Western blot confirmed higher expression of *SPATA6* in rams with larger testicles.

Functional assays validate the importance of hormonal regulators. Han et al. [42] used a DNA vaccine targeting *KISS1*, a neuropeptide that controls GnRH release, resulting in reduced testosterone, testis size, and mating behavior. Early work by McKeown et al. [91] reported that immunization against INH-α (inhibin alpha) expanded testicular volume without improving semen characteristics. More recent analyses under high-altitude Tibetan conditions further showed that inhibin subunit genes *INH-α* and *INH-βA* were down-regulated in GnRH-immunized rams, consistent with reduced inhibin A secretion, while the expression of *INH-βB* remained low across groups. Coordinated declines in *GnRH-R*, *LH-β*, *FSH-β*, *LH-R*, and *FSH-R* expression were also reported, which highlights endocrine regulatory pathways as key molecular candidates influencing ram fertility [92]. Overall, these genes show potential as markers for improving ram fertility through marker-assisted selection.

### 6.3. Genome-Wide and Y-Chromosome Variation

Recent genome-wide association analysis (GWAS) identified 35 QTLs across 16 autosomes significantly associated with semen traits in Merino rams, including ejaculate volume, gross motility, sperm concentration, and percent post-thaw motility [70]. Within 0.5 Mb of significant SNPs, a total of 290 unique candidate genes were discovered. Several genes that have been identified as linked to spermatogenesis and sperm function were highlighted, such as *SLC2A8*, which is involved in sperm glucose transport and motility, *ACTRT2*, a structural protein in the sperm midpiece, *MAPK3*, a regulator of sperm hyperactivation, *SH2B1*, which is linked to motility and metabolic signaling, and *SORD*, which is associated with cryotolerance and post-thaw motility. The presence of genes such as *PADI2*, *RAB3B*, and *ALDOA*, known for their roles in epididymal maturation, acrosome reaction, and conception success, further emphasizes the functional relevance of the identified loci. Overall, even though environments and management factors should be considered, Hodge et al. [70] discovered novel QTLs and functional candidate genes linked to semen traits, which provides a genetic foundation for improving semen quality and fertility in sheep through molecular breeding strategies.

By identifying Y-linked polymorphisms within the male-specific region (MSY) associated with testicular traits, Pei et al. [20] reinforce the idea that developmental genomic variation contributes to fertility outcomes. Specifically, they found that two Y-linked SNPs, *ZFY*16: g.146 C > T and *EIF2S3Y*2 g.77 C > G, respectively, in Suffolk and Hu rams, were significantly associated with larger SC, testicular weight, and epididymis weight, which suggests genetic markers useful for early fertility selection [20]. Additional Y-linked genes, including *DDX3Y*, *ZNF280BY*, and *ZNF280AY*, were also found to have an association with testicular development in Hu sheep [93,94,95]. *ZNF280BY* copy number variation (CNV) and *ZNF280AY* CNV were linked to testicular development in Hu sheep. Their Y-chromosome gene CNVs are negatively correlated with testis size, which may serve as a novel early genetic marker for assessing reproductive capacity in rams [93,94].

Functional evidence from large-animal models indicates that broad regulatory pathways influence postnatal survival and reproductive competence. Davies et al. [96] showed that CRISPR/Cas9-mediated knockout of *IFNAR1/2* in ewes and rams eliminated type I interferon signaling and resulted in developmental abnormalities, high juvenile mortality (~60%), and increased viral susceptibility. Although infertility was observed in ewes, surviving rams remained fertile, suggesting that systemic immune signaling is required for survival to reproductive maturity but is not essential for spermatogenesis itself.

Many gene–trait associations in rams remain breed- or study-specific [28,97], highlighting the need for replication in independent populations. Across studies, transcriptomes map the when and where of fertility-related gene expression, from early testis development to sperm energy output, while genetic variants and CNVs identify which alleles influence these processes in vivo, particularly in the IGF axis, apoptosis/immunity networks, and the male-specific region of the Y chromosome.

## 7. Environment and Management

### 7.1. Hormonal and Nutritional Interventions

Spermatogenesis regulated by hormones is coordinated by the hypothalamic–pituitary–gonadal (HPG) axis, where the hypothalamus secretes gonadotropin-releasing hormone (GnRH), which stimulates the anterior pituitary to release LH and follicle-stimulating hormone (FSH). Spermatogenesis in rams occurs in the seminiferous tubules over about 48 days and begins at puberty (~4 months), when Leydig cells respond to LH by producing testosterone (T) [98]. LH acts on Leydig cells to produce T, while FSH acts on Sertoli cells in conjunction with T to support sperm development by promoting the production of androgen-binding protein (ABP), seminiferous fluid, inhibin, and activin [99]. Several factors negatively affect spermatogenesis including heat stress, season, and nutritional intake. Seasonal changes in photoperiod disrupt neuroendocrine signals, leading to reduced testicular function in many rams. Therefore, it is important to understand the hormonal strategies to support fertility, particularly under stressful or non-breeding conditions [33].

#### 7.1.1. Hormonal Manipulation

Photoperiod is a key regulator of seasonal reproductive activity in rams and their testicular function, hormone secretion, and sexual behavior are all modulated by changes in daylight. Under natural conditions, short day lengths (typical of autumn and early winter) stimulate reproductive activity in rams, while long days (spring and summer) suppress it. Shortening day length (i.e., decreasing photoperiod) stimulates the HPG axis, increasing the secretion of GnRH, LH, and FSH, which increases T production and testicular activity [99]. Melatonin, primarily secreted by the pineal gland in response to darkness, plays an important role in regulating reproductive seasonality in rams by modulating the HPG axis to stimulate the pulsatile release of GnRH, which in turn increases LH, FSH, and T production [100,101,102,103]. In terms of mechanisms, there are two ways of melatonin exerting its effects, receptor-mediated pathways, using MT1 and MT2 receptors found in the hypothalamus, pituitary, testis, and accessory glands [103,104], and direct antioxidant action, neutralizing reactive oxygen species (ROS) and protecting sperm membranes during stress or cryopreservation [105,106,107,108].

Melatonin implants are commonly used to simulate short-day conditions [29,41]. Many studies have demonstrated that exogenous melatonin treatment advances the breeding season, which also increases SC, testicular volume, and plasma T concentrations, and improves sexual behavior indicators such as mount frequency and ejaculatory activity [24,29,32,41,109]. Rekik et al. [41] treated Barbarine rams during late winter and observed a significant increase in SC from 26.7 ± 2.15 cm to 32.1 ± 1.54 cm over 60 days in the melatonin group, compared to 29.5 ± 1.0 cm in untreated controls. Treated rams also showed higher plasma T levels, increased T pulse frequency, and more sexual activity, including mounts and lateral approaches [41]. Similar findings of melatonin implants leading to larger SCs have been observed [32,110]. Cevik et al. [32] showed that melatonin implants led to larger SCs in Charollais rams and increased testicular volume in both Kivircik and Charollais breeds. In the Australian prime lamb industry, a critical production bottleneck occurs during spring mating. Initial studies by Kleemann et al. [110] reported that mixed-age/mature Border Leicester rams treated with melatonin did not improve pregnancy rates in the ewes (90% treated vs. 89% control), but treating young rams (12 months old) with melatonin resulted in significantly higher pregnancy rates in the ewe flock (93% vs. 5%). Exogenous melatonin could dramatically rescue the reproductive performance of young rams in spring, increasing pregnancy rates from 5% to 93%, and melatonin implants significantly increased SC in both young and mixed-age Border Leicester rams over a 42-day period. However, a subsequent 2021 study [7] reported high natural fertility in untreated young rams (82%), resulting in limited additional gains from melatonin treatment (89%), despite significant increases in testicular size, sperm motility, and sexual behavior. At the flock level, melatonin treatment was generally associated with higher pregnancy rates and litter sizes [29,32,109,110], which indicated improvements in overall flock fertility, although one Mediterranean study reported no improvement in lambing rate (94.1% vs. 94.3%) despite increased litter size (1.59 vs. 1.74) [111], suggesting that melatonin primarily mitigates seasonal or maturational constraints rather than enhancing fertility in already optimal rams. In lambs, implants improved T levels and testicular development and reduced variability in sexual maturity [24].

Another alternative to melatonin for stimulating out-of-season reproductive activity could be light manipulation, which is a technique that uses only extra light and can be applied easily. A long-day photoperiod (60 days) after continuous light exposure in Ile-de-France rams, a wool breed, consistently showed a significant increase in testicular volume compared to control rams under natural photoperiods or those receiving only long days [112]. Additionally, Ile-de-France rams subjected to alternating short and long photoperiods at one-month intervals maintained consistently high testicular activity over a period of 2.5 years [23]. Treatment with light manipulation alonecostst less and does not need to use any exoghormonesormone. However, breed-specific effects are present, especially in wool breeds with variations in photo-reactivity, as photoperiod responses are influenced by latitude [113]. Suffolk sheep, a temperate wool breed, responded to seasonal photoperiods at 19°N (Mexico) but not under constant equatorial light (12Light–12Dark) [114,115]. In contrast, Pelibuey sheep (short-haired, native to Mexico) showed individual variations in photo-responsiveness, which indicates some adaptability to seasonal cues despite tropical origins [116]. A summary of the photoperiod and melatonin studies, including breed, latitude, season, dosing or light regimes, outcome measures, and effect sizes, is provided in Supplementary Table S4.

Beyond internal endocrine regulation, exogenous hormonal treatments have been used to modulate reproductive traits in rams, particularly under seasonal or suboptimal breeding conditions. In addition to melatonin, there are another five common hormones that can improve ram fertility, which are kisspeptin (Kp), oxytocin, gonadotropin-releasing hormone (GnRH), equine chorionic gonadotropin (eCG), and Prostaglandin F_2_α (PGF2α) analogs. Kp is essential for initiating puberty and maintaining adult fertility [117], especially its synthetic analog Kp-10, which has shown promising effects on phenotypic fertility traits in rams by activating the HPG axis through stimulation of GnRH secretion, leading to increased LH and FSH release, which support Leydig cell T production and Sertoli cell-mediated spermatogenesis [117,118,119,120,121]. In prepubertal Ossimi rams, Kp-10 treatment significantly increased SC, sperm concentration, sperm motility, and T levels compared to controls [119]. Similarly, weekly intramuscular injections of Kp-10 (5 µg/kg BW) in Ossimi rams for 1 month significantly improved ejaculate volume, sperm motility, viability, rheotaxis, and T levels, with positive effects observed for up to 42 days post-treatment [118]. Additionally, KISS1 and its receptor KISS1R are strongly expressed in the testis of vertebrates [120]. Han et al. [42] developed an antibiotic-free DNA vaccine targeting the *KISS1* gene, which encodes Kps, key upstream regulators of GnRH secretion, to evaluate its potential for immunocastration in ram lambs. Histological analysis confirmed Hu ram lambs vaccinated with the recombinant KISS1-HBsAg-S plasmid showed a strong anti-KISS1 IgG response, which specifically reduces serum T concentrations from days 28 to 98 post-immunization and reduces spermatogenesis [42]. SC and testis weight, length, and breadth were all significantly reduced in vaccinated lambs. Vaccinated lambs showed near-complete suppression of sexual behavior (mounting, sniffing, and butting) compared to controls [42], which is consistent with other species [120]. Evidence from frogs (e.g., *Pelophylax esculentus*) and mice suggests Kp promotes germ cell proliferation and meiosis. KISS1/KISS1R are present in mature sperm across species, with high seminal Kp levels linked to better sperm quality. The Kp may also protect sperm from oxidative stress and support sperm maturation during epididymal transit [120]. The studies overall indicate that Kp positively influences key reproductive traits such as SC, testis size, sperm output, motility, and libido-related hormone profiles in rams, which make Kp a promising hormonal intervention for enhancing ram fertility, particularly during puberty or in seasonal breeding programs.

GnRH is a key neuropeptide in the reproductive axis, stimulating the anterior pituitary to secrete LH and FSH, which regulate T production, testicular function, and spermatogenesis [99]. In rams, exogenous GnRH and its analogs have been used to enhance fertility traits, particularly during the non-breeding season or prior to semen collection [122,123]. In Ossimi rams, GnRH analog (buserelin) administration significantly increased plasma T concentrations and improved testicular blood flow dynamics, indicating enhanced testicular function compared to controls [123]. However, chronic or repeated use of GnRH agonists in human study has shown side effects, potentially suppressing GnRH release and leading to reduced LH and FSH over time [124,125,126]. Therefore, GnRH is best applied as a short-term intervention for enhancing sperm quality, especially under conditions of seasonal suppression or before semen collection.

The eCG hormone exhibits both LH- and FSH-like activity, depending on the tissue, and has been used to modulate reproductive traits in rams, especially during the non-breeding season. Its primary action is to stimulate T production by acting on Leydig cells, while also influencing sexual behavior, testicular development, and potentially sperm quality [99,127,128]. In adult rams, eCG treatment increased plasma T concentrations during both the breeding and non-breeding seasons [127]. It was also associated with enhanced sexual behavior, particularly courtship behaviors and a tendency for more frequent ejaculation, especially during the breeding season. However, the study did not report consistent improvements in sperm quality. Ungerfeld and Bielli [129] reported no effect of weekly eCG administration (0, 100, or 400 IU) on scrotal growth, semen traits, or sexual behavior in prepubertal Milchschaf lambs. Similarly, Beracochea et al. [128] found that although eCG treatment (0, 400, or 700 IU) during the non-breeding season stimulated T secretion in rams, its effects on sperm motility and morphology were limited. Unlike in bucks [130], eCG did not improve main sperm quality traits, which might be due to the dosing interval (6 days vs. 5 days in goats) or faster metabolism of eCG in rams [128]. Some improvements were observed in the cryoresistance of thawed sperm, particularly in motile and progressively motile sperm, while overall sperm quality traits remained unaffected.

Another hormone is oxytocin, which is a neurohormone produced by the posterior pituitary and locally in the male reproductive tract, including Leydig cells and the epididymis, where it does not stimulate spermatogenesis directly but facilitates sperm transport by increasing the contractility of seminiferous tubules and epididymal ducts [131,132,133]. While short-term oxytocin use can enhance ejaculate volume, prolonged use may impair sperm quality, likely due to interference with maturation [131]. Oxytocin and its receptor are important targets for regulating ram fertility, especially in the regulation of ejaculatory efficiency and sperm emission [131]. Prostaglandins (PGs), particularly PGF2α analogs such as cloprostenol and dinoprost, have been investigated in rams to enhance semen quality and sexual behavior, even though research remains limited. These compounds act via smooth muscle contraction in the male reproductive tract, facilitating sperm transport from the epididymis to the vas deferens [134]. When administered prior to semen collection, PGF2α analogs (e.g., cloprostenol and dinoprost tromtehamine) have been shown to improve ejaculate volume, sperm concentration, and mass motility in various species, including rams [135,136,137]. For example, with a treatment of cloprostenol before 30 min of semen collection, rams showed an increase in semen volume, sperm concentration, and total sperm per ejaculate [136]. Cloprostenol (0.15 mg) significantly increased semen volume and mass motility in Katahdin rams [137], while dinoprost (10 mg) improved mating behavior but had a lower efficiency than the cloprostenol-treated group. One study found no significant improvement of sexual behavior with dinoprost in Saint Croix rams [138]. Overall, PGF2α analogs show promise in improving semen parameters and some behavioral traits in rams, while few data are available for rams [139].

Developmental hormonal exposure can also impact testicular traits later in life. For instance, rams prenatally exposed to excess T exhibited significantly reduced SCs and had a lower sperm concentration as adults, despite having normal sexual behavior and hormonal responses to GnRH stimulation [140]. The findings indicate that disruptions to the endocrine environment during gestation can impair testicular development and fertility potential, even when postnatal sexual behavior remains unaffected [140].

Overall, the hormonal regulation of ram reproduction is centrally coordinated by the HPG axis, with key inputs from GnRH, LH, FSH, and T, and is further modulated by environmental and endocrine signals. Without intervention, seasonal changes in photoperiod naturally influence T, thyroid hormones, and semen quality, with peak fertility in late summer to early autumn [26]. Exogenous interventions such as melatonin implants, Kp analogs, GnRH, eCG, oxytocin, and PGF2α analogs have demonstrated potential in enhancing reproductive traits, particularly under seasonal suppression or developmental stages. However, individual variability in response, related to breed, photoperiod sensitivity, and developmental timing, emphasizes the need for targeted application strategies.

#### 7.1.2. Nutritional Management

Nutritional status, particularly energy intake and diet composition, can influence testicular development, hormone production, spermatogenesis, semen quality, and even offspring phenotype in rams.

Energy balance is one of factors that affects testicular development in young rams. Feeding a high-grain (HG) ration to 30-day- to 6-month-old Hu rams significantly accelerated testicular development and hormonal activity compared to hay-fed controls [85]. Specifically, HG-fed rams showed an increase in SC from 11.81 ± 0.40 cm to 13.08 ± 0.38 cm, and epididymal weight increased from 23.62 ± 0.80 g to 29.83 ± 2.13 g. Additionally, serum T levels in the HG group reached 2.09 ± 0.42 ng/mL by day 30, which was more than five times higher than the levels in the hay group (0.39 ± 0.09 ng/mL), suggesting an endocrine response to short-term dietary energy elevation and indicating that even a brief period of enhanced metabolizable energy intake can stimulate reproductive maturation in prepubertal rams by modulating testicular growth and steroidogenic function [85]. Martin [141] showed that a single short-term supplement of 500 g lupin grain increased LH pulse frequency in rams by activating Kp neurons in the arcuate nucleus and dorsomedial hypothalamus, key centers involved in the control of GnRH secretion, which demonstrates a kisspeptin-dependent neuroendocrine pathway through which acute nutritional inputs can rapidly stimulate reproductive hormone secretion, potentially contributing to testicular growth. Conversely, in a nutritional study on Merino rams in Australia, Guan et al. [19] showed that rams on a restricted diet for 65 days had a lower SC, testis weight, sperm production, and Sertoli cell number and quality compared to those on maintenance or high diets. In a follow-up transcriptomic analysis, Guan et al. [18] found that nutritional restriction altered the expression of genes involved in spermatogenesis, apoptosis, and cell cycle regulation. Specifically, modules (M7, M9, and M10) were significantly correlated with traits such as SC. Modules 7 and 9 included genes associated with spermatogenic activity (e.g., *RXFP1*, *ITCH*), apoptosis (e.g., *CFLAR*, *TAF9B*) or both (e.g., *NFKBIL1*, *XRCC5*), while M10 was only composed of apoptosis-related genes (e.g., *MAP3K7*, *EPHA7*). These modules showed strong negative correlations with SC and sperm output, and positive correlations with germ cell apoptosis. Overall, Guan et al. [18,19] indicate that nutrition affects both testicular development and gene expression related to fertility.

The magnitude and duration of energy imbalance determine whether fertility traits are rescued or remain resilient. When Rambouillet rams were forced to gain or lose 12% body weight over 84 days, SC and circulating IGF-1 tracked body condition, and conventional semen traits and mating fertility were stable across planes of nutrition, which is likely due to the loss of fat within the scrotum rather than a loss of actual testicular tissue [142]. This apparent functional resilience does not preclude subtler consequences: lambs sired by the energy-restricted group were approximately 0.3 kg heavier at birth, suggesting paternal nutritional history can exert intergenerational effects even when immediate semen quality appears intact, but paternal effects and their mechanisms remain unclear, especially in livestock species [143]. In contrast, McPherson et al. [144] reported that murine sires on a restricted diet produced male and female offspring with lower birth weights than those from sires on a standard chow diet. Earlier work found no effect of different nutritional planes on libido in Merino rams [145]. Semen traits such as volume, motility, concentration, and morphology did not differ between groups and ewe fertility was unaffected [142].

Furthermore, beyond energy balance, strategic supplementation with vitamins, minerals, and amino acids provides an additional way to improve the overall reproductive success in rams. A 60-day pre-breeding diet including vitamins A, D, and E, along with Zn, Fe, Mn, Cu, Co, I, and Se, increased SC from 32.10 cm to 34.71 cm and raised ejaculate volume from 1.32 mL to 1.47 mL. It also improved the proportion of live sperm from 79.60% to 81.67%, reduced abnormal sperm from 3.82% to 3.57%, and elevated serum T from 2.45 to 2.68 ng/mL by the end of mating. Fertility outcomes also improved, with an increase in the average number of pregnant ewes per ram and the number of lambs born per ram [22]. Weekly vitamin A injections during the first three weeks of life almost doubled sperm density and enlarged paired-testis weight by 56% at eight months, with a higher LH, FSH, and T and stronger testicular antioxidant activity [146]. Feeding L-citrulline at 4–12 g per day for 70 days raised sperm concentration by up to 18%, improved viability, and increased GnRH, LH, FSH and T while elevating antioxidant enzymes in blood and seminal plasma [147]. Multi-omics analyses show that the highest L-citrulline dose also increased seminal pyruvate and amino-acid levels and altered hypothalamic and testicular genes involved in energy and protein metabolism, which may underlie the observed semen improvements [86]. Overall, supplying specific vitamins, minerals and amino acids before breeding or early in life can increase testis size, enhance semen output and quality, and improve flock fertility.

Phytogenic supplementations with *Withania somnifera* (*W. somnifera*) also show an improvement on semen quality and mating behavior [50]. Feeding Blackbelly rams a leftover-feed basal ration supplemented with *W. somnifera* for 40 days showed that the low dose (100 mg kg^−1^ LW) increased ejaculate volume from 0.6 mL to 0.8 mL and SC rose from 31.7 cm to 34.0 cm, whereas the high dose (200 mg kg^−1^ LW) produced ejaculates from 0.5 mL to 0.6 mL, but kept SC the lowest among treatment groups throughout the trial, from 27.4 cm to 29.0 cm. Both doses raised the number of mounts with ejaculation, with the higher dose producing the strongest behavioral response, which suggest that phytogenic supplements can enhance semen output and libido on low-cost diets, but excessive dosing may constrain SC.

Early nutrition has lasting effects on ram fertility. When ewes were fed at only half of maintenance needs from 14 days before to 28 days after mating, the lamb rams showed lower total sperm motility, poorer in vitro fertilization (IVF) blastocyst yield, and more than 200 DNA-methylation changes in sperm [148]. Adding folic acid restored embryo yield but not motility [148]. Paternal diet also is also important. Rams on the negative plane of nutrition (NEG) lost approximately 12% of body weight over 84 days before mating. Lambs sired by NEG rams were significantly heavier at birth than those sired by MAINT (maintenance) rams (*p* = 0.04), and tended to be heavier than those from POS (positive plane) rams (*p* = 0.08) while semen characteristics (volume, sperm concentration, motility, morphology) were not significantly affected by plane of nutrition (*p* ≥ 0.31), except for a minor increase in distal droplets in NEG rams [142], which supports Martin’s [141] review that feeding practices around conception and early life can set long-term limits, or gains, in male reproductive capacity.

Nutrition influences fertility through three overlapping pathways. First, energy shifts trigger fast endocrine changes: a single high-energy meal doubles LH pulse frequency via kisspeptin/neurokinin B/dynorphin (KNDy)–GnRH neurons [141]. Second, diet resets the testicular transcriptome: under- or over-feeding alters thousands of mRNAs, miRNAs and splice variants tied to Sertoli support and apoptosis [18], and L-citrulline re-programs genes for glycolysis and protein turnover along the hypothalamic–testis axis [86]. Third, nutrient status controls redox balance and genome stability: vitamin–mineral or L-citrulline supplements boost antioxidant enzymes [22,147], while feed restriction raises germ cell apoptosis and sperm DNA damage [19] and prenatal undernutrition leaves lasting epigenetic marks [148].

Nutrition plays a key role in ram fertility. Energy restriction reduces sperm quality and testis size, while vitamin, mineral, and amino acid supplements improve semen traits, hormone levels, and scrotal growth. Early-life nutrition, including vitamin A or maternal underfeeding, affects adult fertility through hormonal and epigenetic changes. Overall, nutrition is a practical tool to support ram reproductive performance.

### 7.2. Thermal Influences

While nutritional status can influence testicular function [18], thermal stress poses a greater challenge to sperm production. Heat stress has several negative effects on ram fertility. It interferes with the ability to regulate testicular temperature, damages the process of sperm production, and reduces the quality of ejaculated semen, which may lead to lower chances of successful fertilization [4,40,149,150].

Most sheep breeds have a thermoneutral zone between 5 and 25 °C [151], but some can handle over 30 °C before feeling heat stress, such as Merino rams [4,152]. Exposing Merino rams to 45 °C for 3 h increased scrotal and testicular temperatures by approximately 4–5 °C, while rectal temperature increased by less than 1 °C [153]. Sperm motility declined between days 15 and 35 after exposure, indicating delayed impairment of mid-to-late spermatogenic stages. Experimental heat stress in rams is typically induced by whole-body hyperthermia or localized scrotal insulation. Rams usually maintain testicular temperature between 2 and 8 °C below core body temperature to remain function properly [150,154], and intra- testicular temperatures between 33 and 35 °C when the ambient air temperature is 20 to 30 °C, resulting in a temperature difference of 4 to 6 °C between the rectum and the testis [149]. To maintain the right temperature for sperm production, rams depend on mechanisms such as the tunica dartos and cremaster muscles, the pampiniform plexus, and scrotal sweat glands [149,155,156]. The tunica dartos muscle contracts or relaxes the scrotal skin to adjust surface area and thickness while the cremaster muscle helps lower the testis during heat stress. The pampiniform plexus, locating in the spermatic cord, functions as a countercurrent heat exchanger, cooling incoming arterial blood. The scrotal sweat glands contribute to heat loss through evaporative cooling [149,150,155,156,157]. Additionally, scrotal thermoregulation is tightly controlled by sympathetic adrenergic nerves and adrenal hormones. When scrotal skin temperature exceeds 35 °C, apocrine sweat glands discharge in rhythmic pulses every 2 to 14 min to promote cooling [158]. When the surrounding temperature or direct scrotal heat exceeds the capacity of thermoregulatory systems, the testicles can overheat by 6 to 8 °C [157,159]. Recovery duration depends on the severity of damage, typically 9–11 weeks, but “survivor” rams may recover faster or resist damage entirely [160]. Rams exposed to prolonged desert heat in summer showed an increase in SC and testicular volume [4,21,40], which suggests the mechanism of testicular thermoregulation in heat-stressed male ruminants and is likely due to relaxation of the cremaster muscle [150]. In another review study of ruminants, Rizzoto and Kastelic [158] reported that prolonged heat exposure, such as 28 days of scrotal insulation, leads to reduced T levels and severe disruptions in sperm morphology and testicular structure. Furthermore, local testicular heating results in more pronounced sperm quality impairments than whole-body heat exposure, suggesting that physiological regulation depends on the nature of thermal stress. However, an initial episode of heat stress may induce adaptive mechanisms that mitigate the negative effects of subsequent exposures.

The presence and length of scrotal wool also significantly influence thermal responses [158]. Wool breeds may have higher energy costs for thermoregulation than hair sheep (e.g., Santa Inês), indicating that coat type affects thermoregulatory efficiency [161]. Fully fleeced rams exposed to local testicular heat stress exhibit panting and peripheral vasodilation, whereas sheared rams maintain lower body temperatures and compensate through increased oxygen consumption without panting or vasomotor responses [158]. Rams with short scrotal wool experience acute drops in skin temperature due to rapid sweat evaporation, while wool-covered scrota initially warm up from an exothermic reaction before gradually cooling. These responses are neurologically mediated, as blocking the superior perineal nerves eliminates both sweating and panting [158]. Hair sheep breeds, such as Blackbelly and Pelibuey, have better thermotolerance than meat breeds, such as Dorper and Katahdin, due to skin folds which are associated with testicular blood flow, scrotal surface area, and sweat gland size and density [153,162,163,164,165].

At the cellular level, heat stress interferes with semen quality and spermatogenesis, which likely results in reduced activity of the genes needed for meiosis, increased apoptosis, DNA damage, and the induction of abnormal sperms [8,21,149,160,166]. Heat stress lowers semen quality in rams by affecting hormones, body functions, and metabolism, and by increasing the energy needed to keep a normal body temperature [150,151]. The semen traits most affected are progressive motility, sperm abnormalities, membrane integrity, sperm concentration, and ejaculate volume [150]. These changes are linked to stress-related hormonal changes, especially the rise in cortisol, which suppresses reproductive hormones including GnRH, LH, FSH, and T [151,167,168,169,170,171]. Additionally, heat stress can alter thyroid hormone and insulin levels, further affecting metabolism and overall reproductive function [150,169,172,173,174,175]. In terms of gene expression, structural and DNA damage in sperm often arises from oxidative stress, apoptosis, and reduced testicular blood flow [150]. Five DEGs, *CDK1*, *SYCP2*, *SYCP3*, *DDX4*, *TNP1*, mostly related to meiosis and sperm development, were significantly down-regulated after heat stress [8]. Table 3 provides an overview of the genes influenced by heat stress. Detailed descriptions of the functions with additional genes are provided in Supplementary Table S3. Specifically, compared to Hu rams, Wugu–Hu rams have a stronger transcriptomic response to heat stress, with more than 10,000 DEGs identified and enriched in GO and KEGG pathways related to meiosis, cell cycle, sperm motility, and fertilization [8]. Houston et al. [176] found that heat stress would activate DNA repair pathways and apoptosis in testicular germ cells, which involves genes such as DNA repair genes (e.g., *ERCC1*, *GADD45A*) and oxidative stress response genes (e.g., *HMOX1*, *SOD2*). The damage occured immediately up to 8 days after exposure, which suggests vulnerable early spermatogenic stages [149,176]. In contrast, Alves et al. [160] showed that sperm DNA fragmentation peaks 21 days after heat stress and gradually recovers by day 35, which highlights the delayed impact on ejaculated sperm. Heat stress affects DNA integrity in both developing and mature sperm. However, while DNA damage occurs in ram sperm, it is not a strong predictor of fertility, and Fourier harmonic amplitude (FHA), a shape-based nuclear metric, may offer better predictive value [55]. Ureña et al. [177] examined the effects of seasonal heat stress on the sperm transcriptome in rams. Significant transcriptomic differences were observed between summer (heat-stress conditions) and autumn (comfort conditions) ejaculates, which showed 92 down-regulated genes, such as *ALCAM*, *NOTCH2*, *URI1*, *CCT4*, *PRPF38B*, and *SRSF10*, and 6 up-regulated genes, including *FAM126B*, *PNLIPRP3*, *MSI2*, *RASIP1*, *RGS2*, and *FAM90A1*, under heat-stress conditions. The upregulation of the six genes is primarily associated with stress response, apoptosis regulation, and spermatid development, while several key genes involved in spermatogenesis, sperm motility, membrane stability, and mitochondrial function were down-regulated. GO and pathway analyses further revealed disruptions in oxidative phosphorylation, mitochondrial function, and reproductive system regulation [177].

Abnormalities in sperm after heat stress often occur during the later stages of spermatogenesis, particularly in pachytene spermatocytes and round spermatids. These stages are most sensitive to elevated testicular temperatures and sperm abnormalities (e.g., head defects, midpiece defects, tail coiling) begin to appear 9 to 24 days after heat exposure [149,178]. Even among rams with large SCs, such as Dorper and Katahdin, semen quality tends to be lower compared to heat-adapted breeds [4,21,40]. However, a drop in semen quality does not always limit the fertility of Dorper rams [21,179]. The effects of heat stress on ram reproduction vary across studies and are not consistently reported [150]. The extent of fertility damage depends on how long and how severe the heat exposure is. In mice and rats, even short exposures of scrotal insulation (1.5–4 days) at 32 to 41 °C can reduce motility and sometimes sperm concentration, depending on heat severity and semen collection frequency [149,180]. The impact of heat stress on ram fertility in field conditions is hard to measure because sperm damage appears weeks after exposure, rams vary in heat sensitivity, and fertile rams can mask the poor performance of heat-affected ones during flock mating [149].

Genome-wide analyses suggest that variation in fertility traits is partly shaped by climatic adaptation. An integrated analysis of environmental data and 49,034 SNPs across 32 sheep breeds identified climate-mediated selection of genes related to energy metabolism and endocrine regulation, with *TBC1D12* associated with temperature and precipitation variables [181]. Consistently, transcriptomic studies show that heat stress disrupts pituitary gene expression, including pathways involved in LH and FSH secretion [182].

### 7.3. Biomedical and Pathological Constraints on Fertility

Infectious agents impair ram fertility through mechanisms that determine whether damage is transient or permanent. Viral infections such as Bluetongue virus serotype 3 (BTV-3) can cause severe but reversible azoospermia and oligospermia, with semen quality recovering after one spermatogenic cycle (50–53 days), during which progressive motility increased from 11.3% to 32.5% and normal morphology from 32.5% to 72% [183]. In contrast, *Brucella ovis* (*B. ovis*) induces chronic epididymal inflammation and TGF-β1–mediated fibrosis, resulting in irreversible fertility impairment [184]. Host resistance involves innate immune pathways, with *MyD88* and *TLR9* implicated in infection control and natural variation in *TLR9* associated with reduced disease susceptibility in sheep [185,186]. Other pathogens, including *Actinobacillus seminis* (*A. seminis*), *Histophilus somni* (*H. somni*), and parasitic infections such as *Trypanosoma* spp., also contribute to epididymitis and fertility impairment, particularly in young rams [187,188].

Effective management integrates epidemiological risk factors with accurate diagnostics. Ram-to-ram transmission is common, and older rams (≥6 years) and large flocks (>100 breeding rams) show higher infection risk, supporting age-based segregation strategies [189,190,191]. Serological testing remains widely used but shows variable accuracy depending on cutoff selection, with the IDEXX ELISA (45% threshold) providing the best balance (38.1% sensitivity, 92.0% specificity) [191]. False positives are frequent, with 29% of seropositive rams uninfected on confirmatory testing [192], which emphasizes the value of molecular diagnostics, including species-specific PCR, and genetic screening to prevent hereditary disorders such as junctional epidermolysis bullosa caused by *LAMB3* mutations [193,194].

### 7.4. Social and Behavioral Influences

Ram fertility is shaped by more than just physiological traits. Social context, sexual behavior, and management conditions influence whether a ram successfully mates and fertilizes ewes. These behavioral influences include stress during mating, seasonal libido changes, sexual experience, and dominance rank [30,31,113,195].

Social stress during mating can suppress reproductive success. In Kleemann et al. [110], mixed-age rams that were yarded overnight with ewes on six occasions (i.e., six separate nights) during the first three weeks of the mating period showed significantly lower pregnancy rates (65.5%) compared to rams that remained in paddocks without yarding (89.5%). This reduction occurred despite no observed differences in SC or body condition, indicating that the decline in reproductive performance may be attributed to behavioral suppression, potentially arising from dominance hierarchies among rams or stress responses induced by repeated yarding [196,197]. Studies on sexual behaviors driven by seasonal and photoperiod effects showed reduced libido in young rams during spring and increased mounting activity during the off-season [62,110]. Experience and social rank further influence behavior, with young and less experienced rams showing lower mating success compared to adults [4]. High-ranked lambs reached puberty earlier and showed earlier sexual behavior compared to subordinates, including earlier onset of semen production and mounting behavior [30]. As adults, dominant rams showed more mounting attempts, while subordinates displayed more courtship behaviors and a higher ratio of ejaculations to mounts, suggesting better mating efficiency [31]. However, social ranks are not always related with sexual behaviors and fertility, which depends on various factors, such as seasonal variations, age, rank status (dominance or sub-dominance), individual differences/preferences, or familiarity with other rams.

Management conditions can also affect semen traits. David et al. [62] noted higher motility in semen collected during afternoon sessions, possibly due to social stimulation by seeing other rams being collected in the morning or human intervention. They also found that repeated ejaculation reduced volume and concentration but slightly increased motility [62]. Similarly, Fahey et al. [198] found that exposing rams to ewes in estrus, either through sight and smell or brief physical contact, led to acute increases in semen concentration and volume on the first day of collection. However, these effects were not sustained across repeated daily collections, which suggests that such stimulation has only minor, short-term benefits for semen output and no consistent effect on libido [198].

Overall, environmental and management factors play a strong and often immediate role in ram fertility traits, with varying degrees of impact depending on breed, age, and timing of intervention. Nutritional strategies, mainly via diet and energy-balance control, can impact semen quality. Hormonal treatments involving melatonin were especially effective in photoperiod-sensitive breeds during the non-breeding season, enhancing libido and testicular traits. Heat stress can reduce spermatogenesis and compromise DNA integrity during thermal extremes, while rams have regulatory systems. These findings suggest the integration of targeted management strategies as complementary tools alongside genetic selection to optimize reproductive performance.

## 8. Conclusions

This review summarizes current knowledge on ram fertility in relation to key phenotypic traits, including testicular characteristics, semen traits, and libido. SC is a practical and moderately heritable testicular trait that is consistently associated with higher sperm concentration, motility, and ewe conception rate. Although current fertility evaluations emphasize semen volume, morphology, and motility, SC should continue to serve as a routine selection criterion. At the molecular level, transcriptomic analyses have identified DEGs involved in testis development, spermatogenesis, and sperm function. Key regulators such as *FOXO1*, *YAP1*, *SMAD4*, and *ITGB1* are linked to Sertoli and Leydig cell activity, testis-cord formation, and cell–matrix interactions. In parallel, GWASs have revealed SNPs in genes such as *SLC2A8*, *MAPK3*, and the Y-linked *ZFY16:* g.146 C > T variant, associated with semen quality and testicular growth. These findings highlight the potential of transcriptomic profiling to discover biomarkers and prioritize candidate genes for selection programs. Rapid gene expression–based screening tools for ram fertility have been reported in applied settings, including New Zealand, but peer-reviewed validation and performance metrics remain limited, warranting independent evaluation before broader use. Finally, environment and management factors, such as photoperiod control, hormonal treatments, nutritional strategies, heat-stress mitigation, and disease control, consistently influence ram fertility, although their effectiveness varies with breed, age and season. Hormonal manipulation, such as melatonin implants, yielded significant gains during the non-breeding season, especially in photoperiod-sensitive breeds. These approaches offer an alternative method for long-term genetic selection.

### Take-Home Message and Implications

Genomic and transcriptomic studies have identified candidate genes and pathways related to ram fertility, but many associations remain breed-specific and require replication and functional validation. Improving ram fertility therefore requires integrated approaches that combine phenotypic assessment, molecular evidence, and targeted management. As a narrative review, this synthesis is subject to selection bias, uneven breed representation, and heterogeneous study designs, highlighting the need for standardized fertility metrics and cross-population validation.

## Figures and Tables

**Table 1:** Genes identified as related to ram fertility, growth, and development traits through transcriptomic analysis.

Molecular Approach ^a^	Bioinformatics/Analysis ^b^	Genes/miR ^c^	Breed(s) ^d^	Citation ^e^
RNA-seq; RT-qPCR	DEG analysis; GO/KEGG enrichment; alternative splicing	*FOXO1* (Forkhead box O1), *YAP1* (Yes-associated protein 1), *SMAD4* (SMAD family member 4), *ITGB1* (Integrin β1), *GATA4* (GATA binding protein 4), *SOX9* (SRY-box TF 9), *DDB1* (Damage-specific DNA-binding protein 1)	Southdown sheep × Hu sheep (*n* = 12)	Xi et al., 2024 [77]
RNA-seq	DE miRNA analysis; target prediction (Miranda); GO/KEGG enrichment	*miR-34c*, *miR-21*, *miR-499b*, *SOX9* (SRY-Box Transcription Factor 9) *SMAD4* (SMAD Family Member 4) *YAP1* (Yes1 Associated Transcriptional Regulator), *DOT1L* (DOT1 Like Histone Lysine Methyltransferase)	Southdown sheep × Hu F1 sheep (*n* = 12)	Xi et al., 2025 [84]
RNA-seq	DEG analysis; lncRNA cis-/trans-target analysis; GO/KEGG enrichment	*ABHD2* (Abhydrolase domain-containing 2), *AK1* (Adenylate kinase 1), *CABS1* (Calcium-binding spermatid protein 1), *ROPN1* (Rhophilin-associated tail protein 1), *TEKT3* (Tektin 3), *CCDC39* (Coiled-coil domain 39), *TTC12* (Tetratricopeptide repeat 12), *SEPTIN2*	All rams were sourced from the Bahri-Dagdaş International Agricultural Research Institute in Turkey.	Hitit et al., 2024 [76]
RNA-seq; RT-qPCR	DEG analysis; GO/KEGG enrichment; lncRNA–mRNA co-expression network	*BMP2* (Bone morphogenetic protein 2), *RASGRF2* (RAS guanine-nucleotide releasing factor 2), TSHZ2 *, *ESRRG* (Estrogen-related receptor γ), *GABRP* (GABA-A receptor π subunit), *PAPPA2* (Pregnancy-associated plasma protein A2), *KRT18* (Keratin 18)	Hu sheep (*n* = 12)	Zhang et al., 2017 [85]
RNA-seq	DEG analysis; WGCNA; alternative splicing	*NFKBIL1* (NF-κB inhibitor-like 1), *CFLAR* (CASP8- and FADD-like apoptosis regulator), *MAP3K1* (mitogen-activated protein kinase kinase kinase 1), *MAP3K7* (mitogen-activated protein kinase kinase kinase 7, TAK1)	Merino sheep (*n* = 24)	Guan et al., 2017 [18]
RNA-seq	DEG analysis; GO/KEGG enrichment; metabolomic integration	*GNRH1* (gonadotropin-releasing hormone 1), *PTPN11* (Protein tyrosine phosphatase N11), *KDR* (Kinase insert domain receptor), *JAK2* (Janus kinase 2), *MCM6* (Mini-chromosome-maintenance complex 6), *PRIM1* (DNA primase subunit 1)	Turpan black sheep (*n* = 16)	Zhao et al., 2023 [86]
RNA-seq; RT-qPCR	DEG analysis; GO/KEGG enrichment	*MTNR1A* (Melatonin receptor 1A), *FSHB* (Follicle-stimulating hormone β subunit), *LHB* (Luteinising hormone β subunit), *GNRHR* (gonadotropin-releasing hormone receptor), *AVP* (Arginine-vasopressin), *PDYN* (Prodynorphin), *CGA* (Glycoprotein-hormone α subunit), *GABRD* (GABA-A receptor δ subunit), *TSHB* (Thyroid-stimulating hormone β), *MCHR1* (Melanin-concentrating-hormone receptor 1), *NPY* (Neuropeptide Y), *CRABP1* (Cellular retinoic-acid-binding protein 1)	Rasa Aragonesa sheep (*n* = 59)	Lakhssassi et al., 2023 [87]
RNA-seq	DEG analysis	*MAPK3* (mitogen-activated protein kinase 3, also called ERK1), *MED6* (Mediator Complex Subunit 6), *GTL2* (also known as MEG3, Maternally Expressed Gene 3), *MKLN1* (Muskelin 1), *CHST4* (Carbohydrate Sulfotransferase 4), *LMBR1* (Limb Development Membrane Protein 1), *TGFB2* (Transforming Growth Factor Beta 2), *KRT4* (Keratin 4), *IVL* (Involucrin), *RNF151* (Ring Finger Protein 151), *OXCT2* (3-Oxoacid-CoA transferase 2)	Merino sheep (*n* = 16), Dohne sheep (*n* = 16), and Poll Dorset (*n* = 13)	Hodge et al., 2021 [28]

Note: miR = MicroRNA; RNA-seq = RNA-Sequencing; DEG = Differentially expressed gene; GO = Gene Ontology; KEGG = Kyoto Encyclopedia of Genes and Genomes; WGCNA = Weighted Gene Co-expression Network Analysis. *n* = the number of rams in total including rams not involving in transcriptomic analysis. * Genes with limited information. ^a^ Molecular approach: Experimental methods used to generate molecular data. ^b^ Bioinformatics/analysis: Computational analyses performed to interpret results. ^c^ Genes/miR: Genes or microRNAs identified as significant under the study’s criteria. Only the genes mentioned in the paragraphs are shown. ^d^ Breed(s): Full breed names or crossbred types of sampled rams. ^e^ Citation: Reference for the source study. Additional genes with functions and inferred phenotypic traits affected are provided in Supplementary Table S1.

**Table 2:** List of candidate genes associated with ram fertility using different technologies and methodologies across sheep breeds.

Experimental Technique	Analytical Approach	Genes	Breed(s)	Citation
PCR-RFLP genotyping	Association analysis	*IGF1* (Insulin-like growth factor 1)	Sanjabi sheep (*n* = 96)	Bakhtiar et al., 2017 [17]
		*LEP* (Leptin)		Bakhtiar et al., 2017 [3]
		*CYP19* (Aromatase cytochrome P450) *MTNR1A* (Melatonin receptor 1A)		Kianpoor et al., 2018 [88]
PCR + Sanger sequencing; KASPar SNP genotyping	Association analysis	*IGFALS* (Insulin-like growth factor-binding protein acid-labile subunit)	Hu sheep (*n* = 1662)	Ma et al., 2024 [75]
PCR + Sanger sequencing genotyping	Association with semen traits (FecXR allele)	*BMP15* (Bone morphogenetic protein 15)	Rasa Aragonesa sheep (*n* = 15)	Abecia et al., 2020 [6]
qPCR	Differential expression analysis	*BMPR1B* (Bone Morphogenetic Protein Receptor 1B), *BMP15* (Bone Morphogenetic Protein 15), *GDF9* (Growth Differentiation Factor-9), *Smad1*, *Smad5*, *Smad9* (Mothers Against Decapentaplegic Homolog), *RPL-19* (Ribosomal Protein L19)	Small-tail Han sheep (*n* = 3), Sunite sheep (*n* =3)	Chen et al., 2020 [89]
CDS cloning; qRT-PCR; Western blot	Association analysis	*SPATA6* (Spermatogenesis-associated 6)	Hu sheep (*n* = 340)	Kong et al., 2024 [90]
Active peptide immunization	Endocrine profiling	*INH-α* (Inhibin α subunit)	Suffolk sheep, Texel sheep, Blue-faced Leicester sheep, Dorset sheep (*n* =32)	McKeown et al., 1997 [91]
DNA vaccine immunization	Antibody ELISA; endocrine and histology analysis	*KISS1* (Kisspeptin precursor, KISS1 metastasis-suppressor)	Hu sheep (*n* = 6)	Han et al., 2015 [42]
GnRH vaccine	Endocrine profiling; histology and gene expression analysis	Pituitary genes: *GnRH-R* (gonadotropin-releasing hormone receptor), *LH-β* (Luteinizing hormone beta subunit), *FSH-β* (Follicle-stimulating hormone beta subunit) Testicular genes: *LH-R* (Luteinizing hormone receptor), *FSH-R* (Follicle-stimulating hormone receptor), *INH-α* (Inhibin alpha subunit), *INH-βA* (Inhibin beta A subunit), *INH-βB* (Inhibin beta B subunit; extremely low expression)	Tibetan sheep (*n* = 30)	Han et al., 2015 [92]
SNP chip (Illumina OvineHD) genotyping	GWAS (RepeatABEL)	*SLC2A8* (Glucose transporter 8/GLUT8), *ACTRT2* (Actin-related protein T2), *MAPK3* (mitogen-activated protein kinase 3), *ALDOA* (Fructose-bisphosphate aldolase A), *PADI2* (Peptidyl arginine deiminase 2), *SH2B1* (SH2B adaptor protein 1), *SORD* (Sorbitol dehydrogenase), *RAB3B* (Member RAS oncogene family)	Merino sheep (*n* = 246)	Hodge et al., 2023 [70]
PCR-RFLP Y-SNP genotyping	Association analysis	*ZFY* (Zinc-finger protein, Y-linked), *EIF2S3Y* (Eukaryotic translation initiation factor 2 subunit 3, Y-linked)	Nine sheep breeds (*n* = 956)	Pei et al., 2023 [20]
qPCR CNV assay; RT-PCR expression	Association analysis	*ZNF280BY* CNV (Zinc finger protein 280B-like, Y-linked)	Six introduced sheep breeds and Hu sheep (*n* = 586)	Pei et al., 2022 [93]
qPCR CNV assay	Association analysis	*ZNF280AY* CNV (Zinc finger protein 280A Y-linked, pseudogene)	Eight sheep breeds (*n* = 723)	Liu et al., 2025 [94]
Full-length gene cloning; qPCR expression; PCR-RFLP genotyping	SNP association analysis	*DDX3Y* (DEAD-box helicase 3, Y-linked)	Nine sheep breeds (*n* = 1069)	Zhang et al., 2022 [95]

Note: SNP = Single nucleotide polymorphism; GWAS (RepeatABEL) = Genome-wide SNP association testing with the RepeatABEL package; CDS = Coding DNA sequence; ELISA = Enzyme-linked immunosorbent assay; CNV = Copy number variation; PCR-RFLP = Polymerase chain reaction—restriction fragment length polymorphism. Only the genes mentioned in the paragraphs are shown.

**Table 3:** Candidate genes in rams that change expression under heat stress.

Genes ᵃ	Up-/Down-Regulation Under HS ᵇ	Timing of Expression Changes ᶜ	Technique ᵈ	Breed(s) ᵉ	Citation
*FAM126B* (Family with sequence similarity 126B), *PNLIPRP3* (Pancreatic Lipase-Related Protein 3), *RGS2* (Regulator of G-protein Signaling 2), *RASIP1* (Ras Interacting Protein 1), *MSI2* (Musashi RNA-binding Protein 2), *FAM90A1* (Family with sequence similarity 90 Member A1)	↑	July	RNA-Seq	Manchega (*n* = 40)	Ureña et al., 2022 [177]
*ALCAM* (Activated Leukocyte Cell Adhesion Molecule), *NOTCH2* (Notch Receptor 2), *URI1* (URI1 Prefoldin-like chaperone), *CCT4* (Chaperonin Containing TCP1 Subunit 4), *PRPF38B* (Pre-mRNA Processing Factor 38B), *SRSF10* (Serine/arginine-rich Splicing Factor 10)	↓				
*CDK1* (Cyclin-dependent kinase 1), *SYCP2* (Synaptonemal complex protein 2), *SYCP3* (Synaptonemal complex protein 3), *DDX4* (DEAD-box helicase 4), *TNP1* (Transition protein 1)	↓	Immediately post 3-day scrotal insulation	RNA-Seq; RT-qPCR	Hu sheep (*n* = 6) and Hu × Wugu sheep (*n* = 6)	Chen et al., 2025 [8]

Note: ↑ = Up-regulated under heat-stress conditions; ↓ = Down-regulated under heat-stress conditions. ᵃ Genes: Genes reported to change expression under heat stress. ᵇ Up-/Down-regulation under HS: Direction of differential expression under heat stress. ᶜ Timing of expression changes: Time points or intervals when expression shifts were detected. ᵈ Technique: Methods used to measure expression (for example, RNA-seq or qPCR). ᵉ Breed(s): Sheep breeds included in the study. *n* is the number of rams in total. The functions of genes can be found in Supplementary Table S3.

## Data Availability

The original contributions presented in this study are included in the article/Supplementary Materials. Further inquiries can be directed to the corresponding author.

## Supplementary Materials

The following supporting information can be downloaded at https://www.mdpi.com/article/10.3390/genes17020210/s1, Table S1: Genes related to ram fertility through transcriptomic analysis. Table S2: Candidate genes related to ram fertility. Table S3: Genes in rams that change expression under heat stress (fertility-related). Table S4: Photoperiod and melatonin studies in rams, with breed, latitude, seasonal context, dosing or light regimes, and fertility-related outcomes.

## Abbreviations

The following abbreviations are used in this manuscript:

ABP	Androgen-Binding Protein
*A. seminis*	*Actinobacillus seminis*
AGID	Agar Gel Immunodiffusion
AI	Artificial Insemination
AS	Alternative Splicing
ASMA	Computer-Assisted Sperm Head Morphometry Analysis
BTV-3	Bluetongue Virus Serotype 3
*B. ovis*	*Brucella ovis*
cAMP	Cyclic Adenosine Monophosphate
CBBP	Community-Based Breeding Program
CDS	Coding DNA Sequence
CFLAR	CASP8- and FADD-Like Apoptosis Regulator
CNV	Copy Number Variation
DA	Differentially Abundant
DEG	Differentially Expressed Gene
EBV	Estimated Breeding Value
eCG	Equine Chorionic Gonadotropin
ELISA	Enzyme-Linked Immunosorbent Assay
ErbB	Erythroblastic Leukemia Viral Oncogene B
FHA	Fourier Harmonic Amplitude
FoxO	Forkhead Box O
FSH	Follicle-Stimulating Hormone
GH	Growth Hormone
GnRH	Gonadotropin-Releasing Hormone
GO	Gene Ontology
GWAS	Genome-Wide Association Study
HG	High-Grain (diet)
*H. somni*	*Histophilus somni*
HPG	Hypothalamic–Pituitary–Gonadal
IGF	Insulin-Like Growth Factor
IVF	In Vitro Fertilization
JEB	Junctional Epidermolysis Bullosa
KEGG	Kyoto Encyclopedia of Genes and Genomes
KNDy–GnRH neurons	Kisspeptin, Neurokinin B, Dynorphin–Gonadotropin-Releasing Hormone Neurons
Kp	Kisspeptin
LAMB3	Laminin Subunit Beta-3
LH	Luteinizing Hormone
lncRNAs	Long Non-Coding RNAs
MAINT	Maintenance
MAPK	Mitogen-Activated Protein Kinase pathway
miR	MicroRNA
MSY	Male-Specific Region of the Y Chromosome
MXE	Mutually Exclusive Exons
NEG	Negative Plane Of Nutrition
NFKBIL1	NF-κB Inhibitor-Like 1
NOM	Number of Mounts
NVSL	National Veterinary Services Laboratories
PCR-RFLP	Polymerase Chain Reaction-Restriction Fragment Length Polymorphism
PGs	Prostaglandins
PI3K-Akt	Phosphoinositide 3-Kinase–Akt pathway
POS	Positive Plane
QTL	Quantitative Trait Locus
RI	Intron Retention
rMATS	Multivariate Analysis of Transcript Splicing
ROS	Reactive Oxygen Species
RT	Reaction Time
RT-PCR	Reverse Transcription Polymerase Chain Reaction
RT-qPCR	Reverse Transcription Quantitative Polymerase Chain Reaction
RXFP1	Relaxin/Insulin-Like Peptide Receptor 1
SC	Scrotal Circumference
SE	Skipped Exon (Alternative Splicing Event)
SNP	Single Nucleotide Polymorphism
T	Testosterone
TGF-β	Transforming Growth Factor Beta
WGCNA	Weighted Gene Co-expression Network Analysis
